# Diagnostic measures comparison for ovarian malignancy risk in Epithelial ovarian cancer patients: a meta-analysis

**DOI:** 10.1038/s41598-021-96552-9

**Published:** 2021-08-27

**Authors:** Arpita Suri, Vanamail Perumal, Prajwal Ammalli, Varsha Suryan, Sanjiv Kumar Bansal

**Affiliations:** 1 Department of Biochemistry, SGT Medical College Hospital and Research Institute, Gurugram, Haryana 122505 India; 2 Department of Obstetrics and Gynaecology, All India Institute of Medical Sciences, New Delhi, India; 3 Department of Biochemistry, Institute of Liver and Biliary Sciences, D1 ILBS Road, Vasant Kunj, New Delhi, Delhi 110070 India

**Keywords:** Biochemistry, Cancer, Immunology, Biomarkers, Diseases, Oncology

## Abstract

Epithelial ovarian cancer has become the most frequent cause of deaths among gynecologic malignancies. Our study elucidates the diagnostic performance of Risk of Ovarian Malignancy Algorithm (ROMA), Human epididymis secretory protein 4 (HE4) and cancer antigen (CA125). To compare the diagnostic accuracy of ROMA, HE-4 and CA125 in the early diagnosis and screening of Epithelial Ovarian Cancer. Literature search in electronic databases such as Medicine: MEDLINE (through PUBMED interface), EMBASE, Google Scholar, Science Direct and Cochrane library from January 2011 to August 2020. Studies that evaluated the diagnostic measures of ROMA, HE4 and CA125 by using Chemilumincence immunoassay or electrochemiluminescence immunoassay (CLIA or ECLIA) as index tests. Using the Quality Assessment of Diagnostic Accuracy Studies (QUADAS-2). We included 32 studies in our meta-analysis. We calculated AUC by SROC, pooled estimated like sensitivity, specificity, likelihood ratio, diagnostic odds ratio (DOR), Tau square, Cochran Q through random effect analysis and meta-regression. Data was retrieved from 32 studies. The number of studies included for HE4, CA125 and ROMA tests was 25, 26 and 22 respectively. The patients with EOC were taken as cases, and women with benign ovarian mass were taken as control, which was 2233/5682, 2315/5875 and 2281/5068 respectively for the markers or algorithm. The pooled estimates of the markers or algorithm were sensitivity: ROMA (postmenopausal) (0.88, 95% CI 0.86–0.89) > ROMA (premenopausal) 0.80, 95% CI 0.78–0.83 > CA-125(0.84, 95% CI 0.82–0.85) > HE4 (0.73, 95% CI 0.71–0.75) specificity: HE4 (0.90, 95% CI 0.89–0.91) > ROMA (postmenopausal) (0.83, 95% CI 0.81–0.84) > ROMA (premenopausal) (0.80, 95% CI 0.79–0.82) > CA125 (0.73, 95%CI 0.72–0.74), Diagnostic odd’s ratio ROMA (postmenopausal) 44.04, 95% CI 31.27–62.03, ROMA (premenopausal)-18.93, 95% CI 13.04–27.48, CA-125-13.44, 95% CI 9.97–18.13, HE4-41.03, 95% CI 27.96–60.21 AUC(SE): ROMA (postmenopausal) 0.94(0.01), ROMA (premenopausal)-0.88(0.01), HE4 0.91(0.01), CA125-0.86(0.02) through bivariate random effects model considering the heterogeneity. Our study found ROMA as the best marker to differentiate EOC from benign ovarian masses with greater diagnostic accuracy as compared to HE4 and CA125 in postmenopausal women. In premenopausal women, HE4 is a promising predictor of Epithelial ovarian cancer; however, its utilisation requires further exploration. Our study elucidates the diagnostic performance of ROMA, HE4 and CA125 in EOC. ROMA is a promising diagnostic marker of Epithelial ovarian cancers in postmenopausal women, while HE4 is the best diagnostic predictor of EOC in the premenopausal group. Our study had only EOC patients as cases and those with benign ovarian masses as controls. Further, we considered the studies estimated using the markers by the same index test: CLIA or ECLIA. The good number of studies with strict inclusion criteria reduced bias because of the pooling of studies with different analytical methods, especially for HE4. We did not consider the studies published in foreign languages. Since a few studies were available for HE4 and CA125 in the premenopausal and postmenopausal group separately, data were inadequate for sub-group analysis. Further, we did not assess these markers' diagnostic efficiency stratified by the stage and type of tumour due to insufficient studies.

## Introduction

Ovarian cancer accounts for the eighth most common cause of deaths globally^1^. Epithelial ovarian cancer (EOC) is one of the most common malignancies affecting perimenopausal women^2^. In India, it is the third leading cause of cancer in females, with a 5-year cumulative estimate of 80,422 cases^1^.

The lack of specific symptoms, effective screening and diagnostic techniques makes ovarian cancer challenging to diagnose in the early stages. Therefore, late-stage diagnosis with metastasis results in a low survival rate. However, the survival rate increases to 92% when the tumor is diagnosed when localised^3^. Therefore, ovarian cancer research's primary goal is to develop a practical, valid screening test to detect the disease at an early stage. The most widely used screening tests are serum cancer antigen (CA125) estimation and Transvaginal sonography^4^, which are non-specific.CA125 is an antigenic determinant recognised by a monoclonal antibody raised as an immunogen using ovarian cell line^5^. Though serum CA125 was initially reported in patients with ovarian cancer, higher levels were also seen during menstruation, early pregnancy, and endometriosis^5^. It is also less sensitive as it is raised in only 80% of all epithelial ovarian cancers (EOC) and only 50% of stage I EOC^6^. Another biomarker that is currently available in the screening and diagnosing EOC is serum Human epididymis secretory protein 4 (HE4), which was approved by the food and drug administration (FDA) in 2008^7^. It is a member of the Wey acidic protein family^8^ and is expressed in healthy tissues of the reproductive and respiratory tract. It is postulated that HE4 is better than CA125 in diagnosing patients with EOC due to high specificity. However, HE4 increases with age, smoking and renal diseases^9^. There are several methods to estimate serum CA125 and serum HE4, such as Enzyme-linked immunosorbent assay (ELISA), Radioimmunoassay (RIA), Chemiluminiscence immunoassay (CLIA) and Electrochemiluminesce immunoassay (ECLIA). CLIA and ECLIA have more sensitivity compared to other tests^10^.

Risk of Ovarian Malignancy Algorithm (ROMA) is a qualitative algorithm that includes the results of serum HE4, serum CA 125 and menopausal status into its value^11^. ROMA's clinical utility to assess the risk of EOC in patients with pelvic mass has been evaluated. In 2008, Moore et al. suggested that the combination of CA125 and HE4 had the highest sensitivity compared to the single marker^12^. In the year 2011, Montagnana et al. concluded that ROMA had superior diagnostic performance in estimating the risk of EOC in premenopausal women^13^. But, ROMA's diagnostic performance compared to CA125 and HE4 is still controversial as the individual studies are affected by limited sample size and random fluctuations. Thus, our study aimed to analyse ROMA's performance by conducting a meta-analysis of the studies using high sensitivity immunoassays like CLIA and ECLIA as the index test. The included studies had CLIA/ECLIA as the index test, which reduced bias due to studies with different analytical methods. This would help in elucidating the performance of ROMA in diagnosing EOC in comparison to other serum markers.

## Materials and methods

### Inclusion and exclusion criteria

We included the studies if (1) CLIA or ECLIA estimated the values of serum HE4 and serum CA125; (2) studies investigated serum HE4 or CA125 or calculated the ROMA for the EOC diagnosis; (3) blood samples were collected before initiation of anti-tumour therapy; (4) studies used the histopathological diagnosis as the gold standard for assessing EOC; (5) enough data could be extracted for the fourfold table. We excluded the studies if (1) control group contained healthy individuals and borderline cases (2) analytical method used was ELISA (3) studies showed prognostic or post-chemotherapy changes in the marker (4) language of the abstract or full paper was in any language except English, and (5) Studies, which calculated sensitivity at fixed specificity using Receiver operator characteristics curve (ROC).

#### Data extraction

We extracted data from the selected studies such as author, publication year, country, study design, detection methods, number of patients, sensitivity, specificity and cutoff value. We chose the one that offered the best test performance for the study with more than one cutoff value. For example, in several detection methods used in one study, we chose the results of the most sensitive method, CLIA.

#### Index tests and reference standard

We calculated the ROMA index using the following formulae.

The premenopausal calculation formula of the ROMA index was: $12+\left(2.38*\mathrm{ln}\left(\mathrm{HE}4\right)\right)+(0.062*\mathrm{ln}\left(\mathrm{CA}125\right))$

The postmenopausal calculation formula of the ROMA index was: 8.09 + (1.04*ln(HE4)) + (0.732*ln(CA125)).

Since different Index tests have different sensitivity, we included only CLIA and ECLIA as the index test to reduce the bias because of pooling studies. We considered the result of the histopathological diagnosis as the reference standard. The criteria from FIGO was taken as the reference for surgical staging. PRISMA flow diagram describes the number of studies screened and included for meta-analysis.

#### Methodological quality assessment

Using the Quality Assessment of Diagnostic Accuracy Studies (QUADAS-2), two authors (AS and PA) independently assessed the methodological quality of the included studies and extracted data using Rev Manager software. In case of conflict between the authors, the discussion helped in reaching a uniform conclusion.

### Statistical analysis

We developed a database on individual study details using a Microsoft Excel spreadsheet and subjected it to statistical analysis using Meta-Disc software version 1.4, a freely available open-source programme. Adding a value of 0.5 could avoid zero value in the study results, and a two-stage analysis was performed. We calculated summary statistics such as sensitivity, specificity, positive and negative likelihood ratios (LR) for each study in the first stage. In the second stage, we estimated overall test accuracy indexes as the weighted average of the summary statistics. Since summary measures (sensitivity and specificity, or LR_+_ and LR_−_) are paired and often inter-related, the apt measure useful in meta-analysis is the diagnostic odds ratio (DOR). The DOR suggests how much higher the odds of having the disease are for the people with a positive test result than those with a negative test result. For each summary measure, we calculated 95% exact confidence limits. We examined the degree of variability (heterogeneity) among the study results by plotting the summary measures on a forest plot. The sampling error variability is likely to be high in a meta-analysis for studies with different sample sizes. Other sources of variation could be due to the study subjects' characteristics, mode of treatment, and design quality. Therefore, assessing the heterogeneity in meta-analysis is crucial because the presence versus the absence of true heterogeneity (between studies variability) can affect the statistical model. Therefore, we calculated inconsistency (I^2^) **statistics** in percentage values along with Tau-square, which suggests the degree of heterogeneity. Value of an I^2^-statistic higher than 50% considered to have significant heterogeneity. We used a random-effect model for calculations because of variation evidence. Since the studies included might have used directly or implicitly different thresholds/cutoff values to define positive and negative results, these cutoff values are likely to be an essential source of variation in DOR. We carried out receiver operating characteristics (ROC) analysis to see if a threshold effect exists**.** If the points in the ROC plot show a curvilinear pattern, there is evidence of threshold effect.

Further, we tested the threshold effect based on the Spearman correlation coefficient between sensitivity (true positive rate) and false positive rate (FPR). A significant inverse correlation could confirm the existence of the threshold effect. Combining study results in these cases involves fitting a ROC curve rather than pooling sensitivities and specificities or likelihood ratios. While establishing homogeneity, likelihood ratios and diagnostic odds ratios could be pooled by the Mantel–Haenszel method (fixed effects model); in case of heterogeneity, we used the DerSimonian Laird method (random-effects model) to incorporate variation among studies. The DOR’s or LR’s were averaged with the Mantel–Haenszel method, whereas, by adopting the DerSimonian Laird method, we obtained the average value of logs of DOR or LR’s. Publication bias between the studies was tested by carrying out a regression analysis of ln(DOR) against the inverse of the square root of effective sample size (1/sqrt(ESS)), with p < 0.05 for slope coefficient suggesting significant asymmetry. We carried out all these analyses following the guidelines given by an earlier publication^14^.

### Meta-regression

In case of great variation detected from the analysis detailed above, we explored reasons for such variations by relating covariates such as the age of subjects and cutoff values. To study the sources of variations in the studies, we used the Moses–Shapiro–Littenberg method. We considered the variable ln(DOR) as the dependent variable and the variables such as age, cutoff value and threshold effect as covariates. The antilogarithm transformations of the resulting estimated parameters were a relative DOR (RDOR) of the corresponding covariate, which suggests the change in the test understudy's diagnostic performance for each unit increase in the covariate. We decided the statistical significance of the coefficient based on a p value < 0.05.

## Results

### Baseline characteristics of the study population

**Figure 1:**
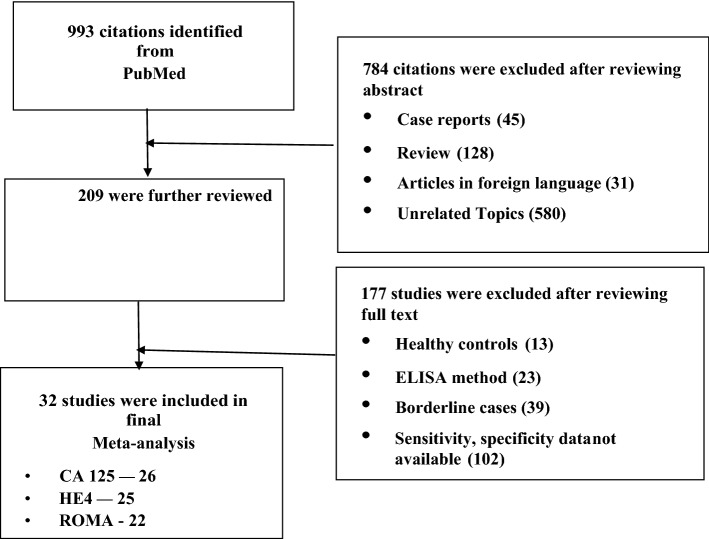
PRISMA Flow-diagram.

**Figure 2:**
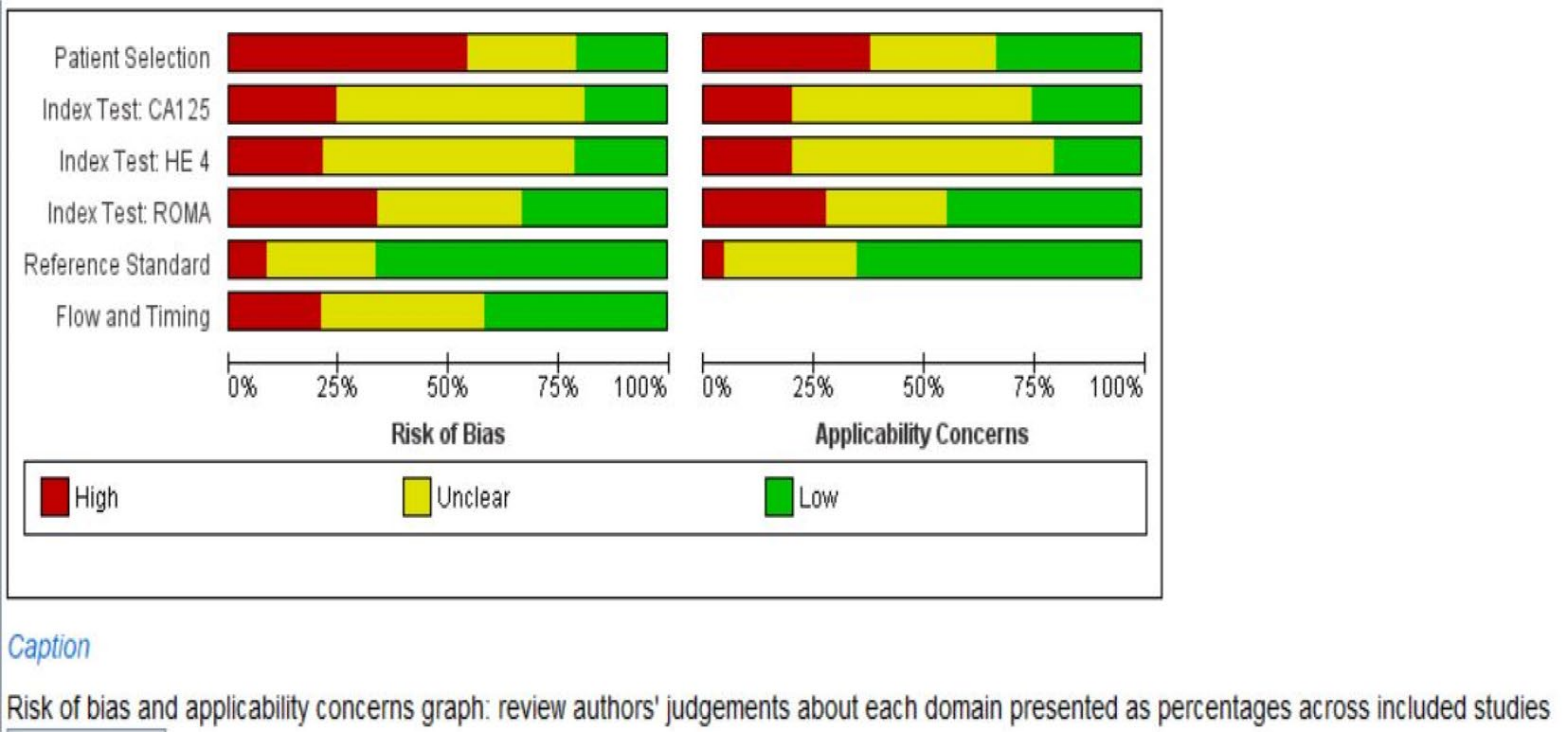
Quality of publications (**QUADAS-2**) on six domains prepared by using Revman 5.3

We searched the studies that were published from January 2011 to August 2020. PRISMA flow-diagram (Fig. 1) shows the stages of the studies screened and included for the analysis, and 32 studies^15–46^ met the inclusion criteria. The number of studies included for HE4, CA125 and ROMA tests were 25^16–19,22–32,34,35,37–43^, 26^16–20,22–35,37–41,43^ and 22^15–18,20,21,26–29,31,34–43,46^, respectively. The patients with EOC were taken as cases, and women with benign ovarian mass were taken as control, which was 2233/5682, 2315/5875 and 2281/5068, respectively, for the markers or algorithm (Table 1). The average age of cases and controls were 54 and 43 years, respectively. Stage-specific distribution showed that about 31% of the EOC cases were with stage III, followed by stage IV (~ 14%). Similarly, about 35% EOC cases were serous type followed by endometroid (~ 11%). We assessed each study's quality on six domains and have shown the overall risk of biases in the QUADAS-2 graph (Fig. 2). About 75% of the studies included showed a low risk of bias for selecting the reference standard.

**Table 1:** Characteristic of patients undergone different diagnostic tests.

Characteristics	HE4 (studies = 25)	CA125 (studies = 26)	ROMA-all (studies = 22)	ROMA (pre-menopause) (studies = 24 )	ROMA (post-menopause) (studies = 26)
Ovarian cases	2233	2315	2281	902	1902
Healthy control	5682	5875	5068	4351	2144
Weighted average age (years)-cases	53.7	53.5	54.5	Not reported	
Weighted average age (years)—control	43.2	43.5	43.1	Not reported	
**Stage specific distribution (%)**					
I	290 (13.0)	294 (12.7)	320 (14.0)	Not reported	
II	93 (4.2)	101 (4.4)	101 (4.4)		
III	691 (30.9)	721 (31.1)	754 (33.1)		
IV	315 (14.1)	342 (14.8)	265 (11.6)		
Not available	844 (37.8)	857 (37.0)	841 (36.9)		
**Type specific distribution (%)**					
Serous	799 (35.8)	819 (35.4)	798 (35.0)	Not reported	
Mucinous	150 (6.7)	150 (6.5)	129 (5.7)		
Endometroid	263 (11.8)	253 (10.9)	242 (10.6)		
Clear	99 (4.4)	95 (4.1)	94 (4.0)		
Mixed	38 (1.7)	38 (1.6)	38 (1.7)		
Others	13 (0.6)	13 (0.6)	11 (0.5)		
Not available	871 (39.0)	947 (40.9)	969 (42.5)		

Human epididymis secretory protein 4 (HE4), Cancer antigen (CA125) and Risk of Ovarian Malignancy Algorithm (ROMA).

### Comparison of diagnostic measures

**Figure 3:**
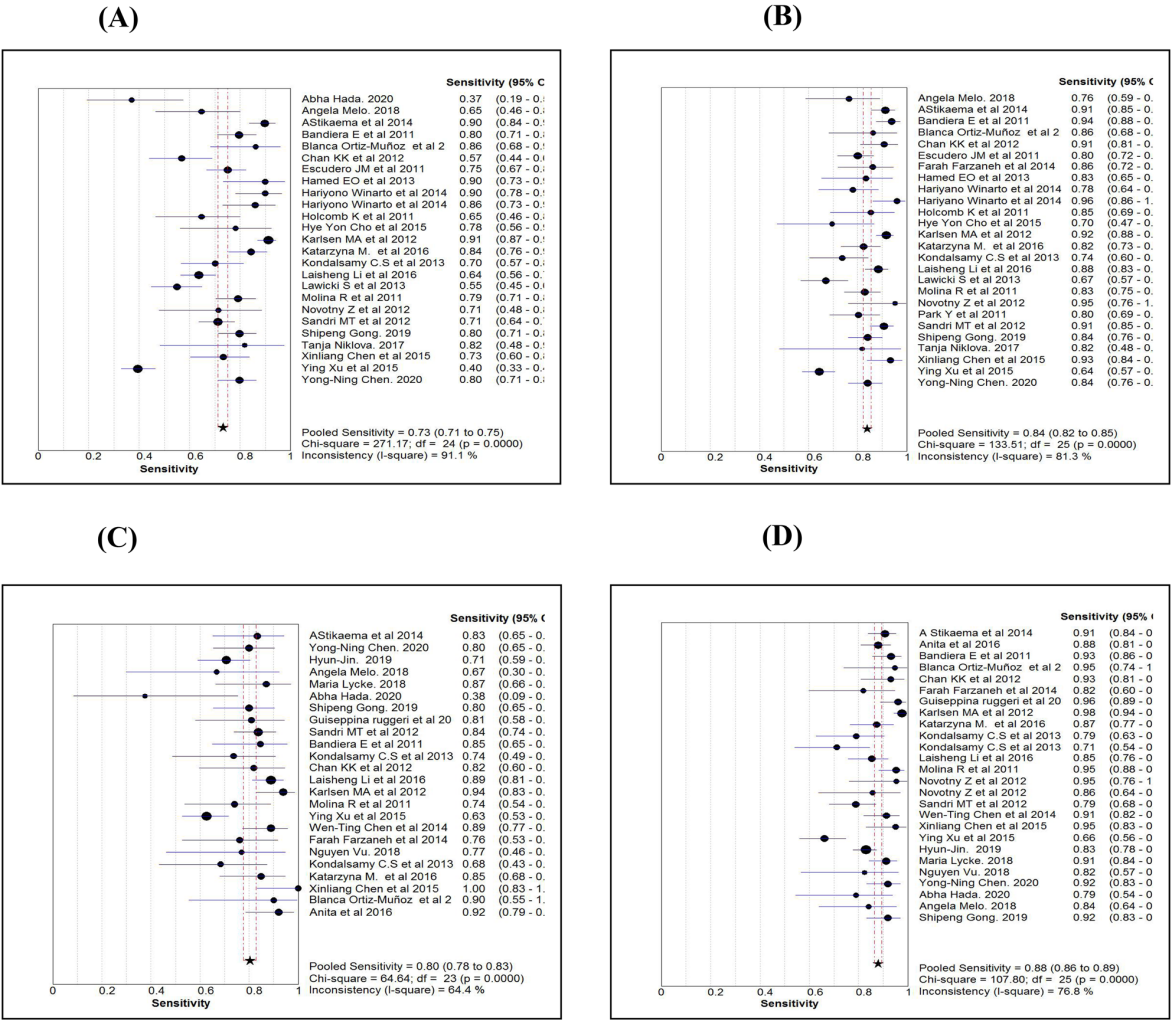
Comparison of Sensitivity estimates of HE4 (**A**), CA125 (**B**), ROMA for pre-menopause (**C**) and ROMA for post-menopause (**D**).

**Figure 4:**
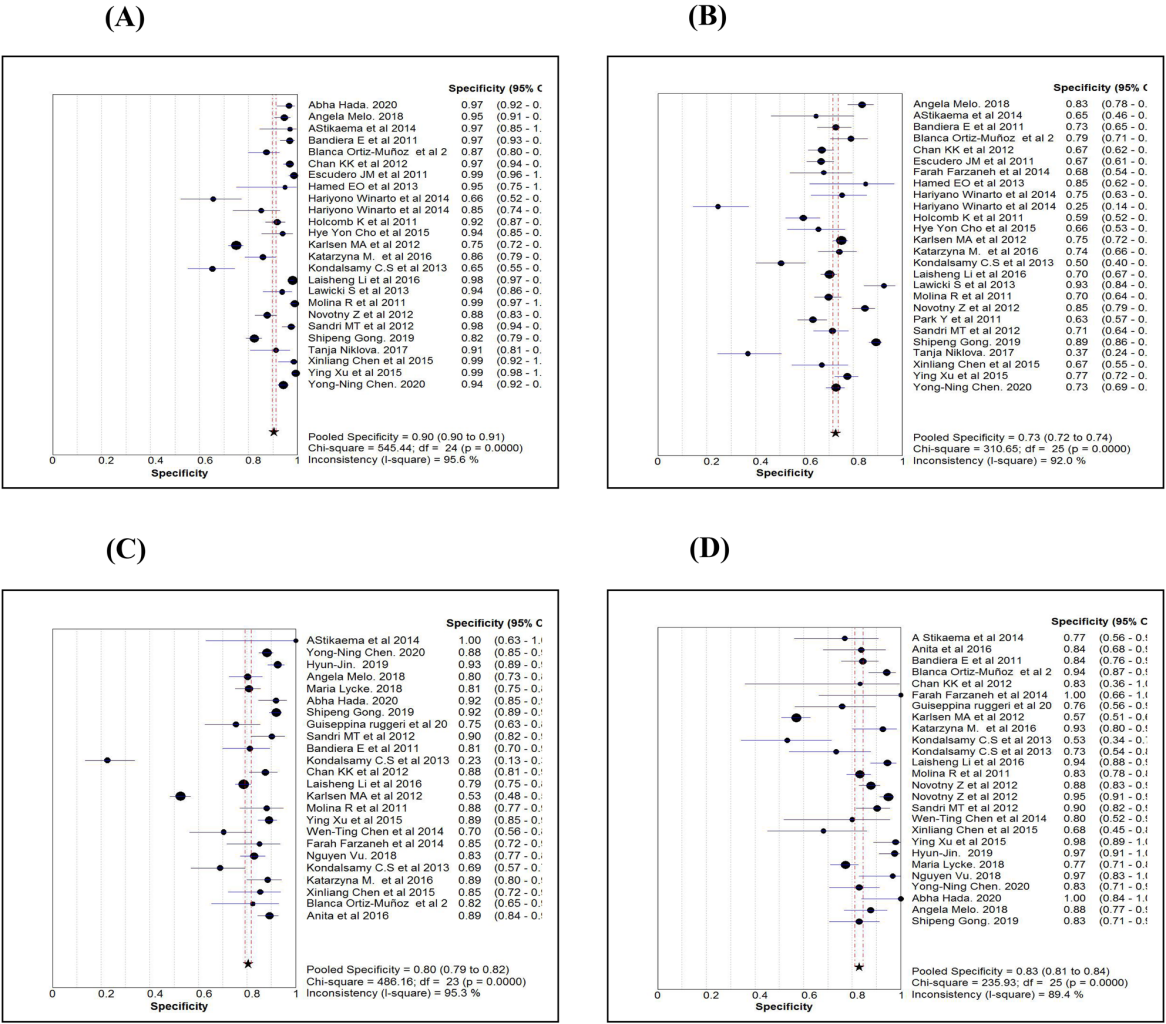
Comparison of Specificity estimates of HE4 (**A**), CA125 (**B**), ROMA for pre-menopause (**C**) and ROMA for post-menopause (**D**).

Table 2 shows the various diagnostic tests calculated for the three markers or algorithm and pooled estimates of each measure. While comparing the sensitivity measures (Figs. 3A–D), the overall sensitivity for HE4 was 0.73 (95% CI 0.71–0.75), significantly less than CA125 and ROMA. In contrast to the sensitivity measure, the overall specificity for HE4 (0.90; 95% CI 0.89–0.91) was significantly higher than the other two markers (Fig. 4A–D). While comparing the diagnostic measures between premenopausal and postmenopausal women using ROMA, we noted that both the sensitivity and specificity levels were more than 0.80 among postmenopausal women and were significantly higher than those measured among premenopausal women.

**Table 2:** Comparison of diagnostic measures by three serum markers.

Procedure	Statistics (95% CI)	Name of marker		ROMA		
		HE4	CA 125	ROMA All	ROMA Pre-Menopause	ROMA Post-Menopause
	Sensitivity	0.73 (0.71–0.75)	0.84 (0.82–0.85)	0.86 (0.84–0.87)	0.80 (0.78–0.83)	0.88 (0.86–0.89)
	Specificity	0.90 (0.89–0.91)	0.73 (0.72–0.74)	0.79 (0.78–0.80)	0.80 (0.79–0.82)	0.83 (0.81–0.84)
Random effect	LR + ve	10.61 (7.28–15.45)	2.91 (2.47–3.42)	4.66 (3.41–6.35)	4.59 (3.41–6.18)	5.86 (4.35–7.89)
	LR –ve	0.27 (0.21–0.34)	0.23 (0.19–0.29)	0.18 (0.13–0.24)	0.26 (0.21–0.33)	0.15 (0.12–0.19)
	Diagnostic Odds ratio	41.03 (27.96–60.21)	13.44 (9.97–18.13)	27.48 (18.70–40.38)	18.93 (13.04–27.48)	44.04 (31.27–62.03)
	Tau-square (%)	67.71	41.90	65.74	51.61	36.19
	Cochrane-Q	107.60 (P = 0.001)	106.84 (P = 0.001)	117.96 (P = 0.001)	67.63 (P = 0.001)	49.54 (P = 0.002)
	Spearman’s correlation	0.49 (P = 0.013)	0.12 (P = 0.576)	0.30 (P = 0.183)	0.18 (P = 0.390)	0.46 (P = 0.019)
Fixed effect	LR + ve	7.73 (7.05–8.46)	2.98 (2.84–3.12)	4.07 (3.84–4.32)	4.30 (3.97–4.66)	4.64 (4.19–5.12)
	LR –ve	0.30 (0.28–0.32)	0.23 (0.21–0.25)	0.18 (0.16–0.20)	0.25 (0.22–0.29)	0.15 (0.13–0.17)
	Diagnostic Odds ratio	37.31 (31.63–44.01)	13.57 (11.93–15.42)	26.24 (22.46–30.65)	19.09 (15.61–23.35)	42.42 (33.78–53.27)
	Tau-square	77.70	76.60	82.20	66.00	49.50
	Cochrane-Q	107.60 (P = 0.001)	106.84 (P = 0.001)	117.96 (P = 0.001)	67.63 (P = 0.001)	49.54 (P = 0.002)
Symmetrical ROC (SROC)	AUC (SE) Q* index (SE)	0.91 (0.01) 0.84 (0.01)	0.86 (0.02) 0.79 (0.02)	0.91 (0.01) 0.84 (0.01)	0.88 (0.01) 0.81 (0.01)	0.94 (0.01) 0.87 (0.01)

Human epididymis secretory protein 4 (HE4), Cancer antigen (CA125) and Risk of Ovarian Malignancy Algorithm (ROMA). Positive Likelihood ratio (LR + ve), Negative Likelihood ratio (LR–ve), Standard error (SE).

**Figure 5:**
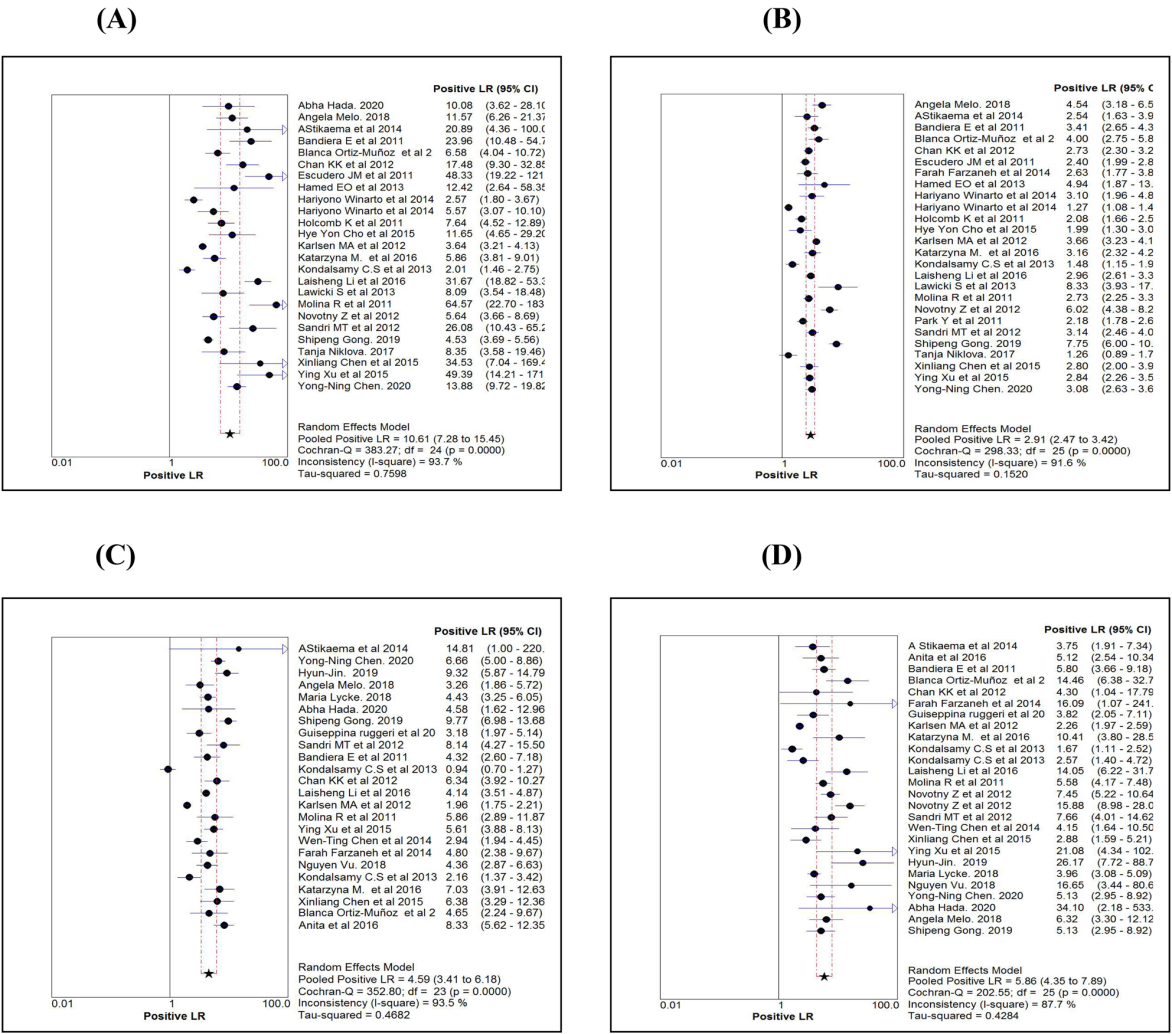
Comparison of positive likelihood ratio estimates (using Random effect model) of HE4 (**A**), CA125 (**B**), ROMA for pre-menopause (**C**) and ROMA for post-menopause (**D**).

**Figure 6:**
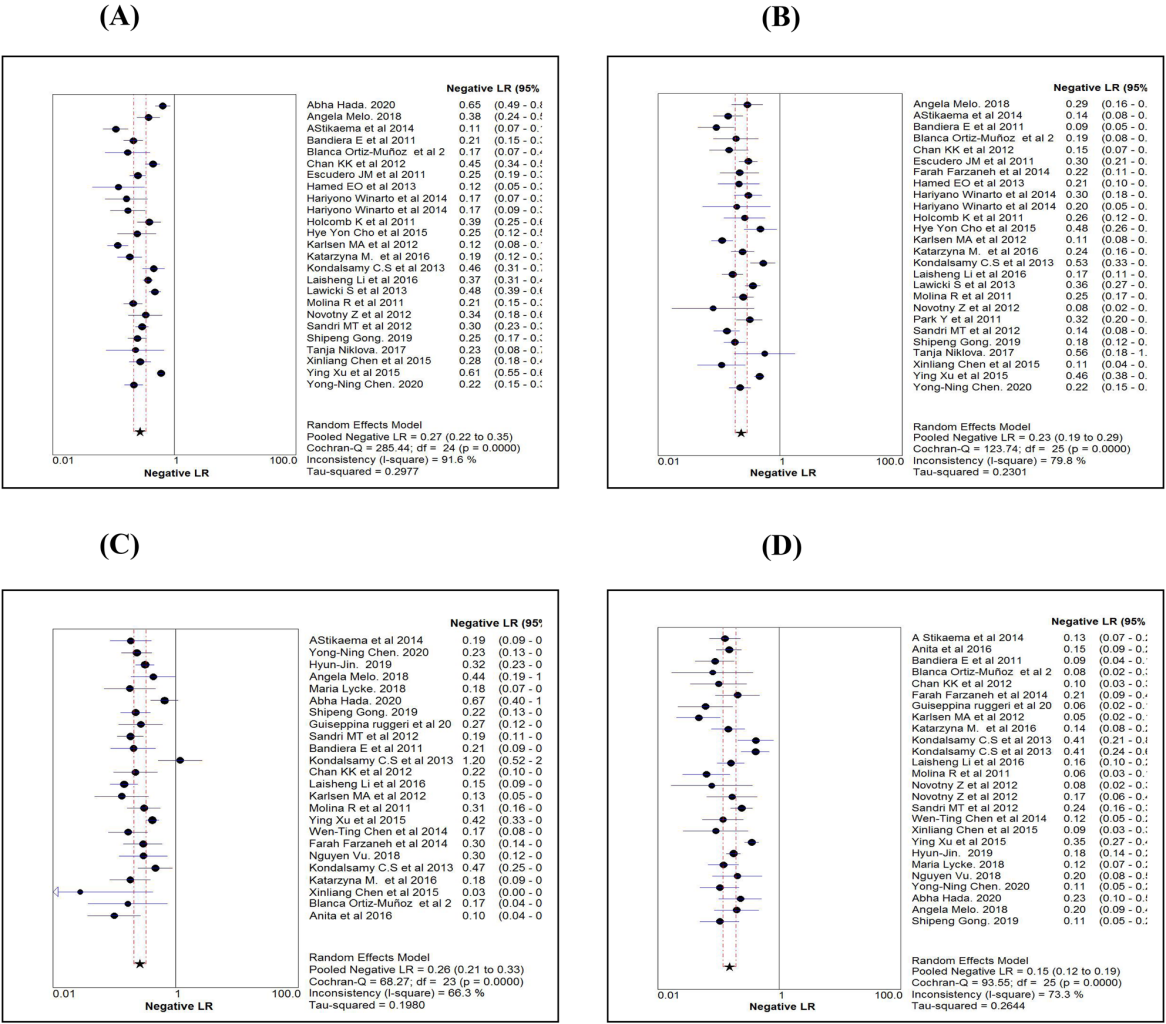
Comparison of negative likelihood ratio estimates (using Random effect model) of HE4 (**A**), CA125 (**B**), ROMA for pre-menopause (**C**) and ROMA for post-menopause (**D**).

The likelihood ratio is another way of looking at the reliability of the test. LR expression has two forms. LR + (the probability of a person who has the disease testing positive divided by the probability of a person who does not have the disease testing positive) and LR- (the probability of a person who has the disease testing negative divided by the probability of a person who does not have the disease testing negative). Comparison of pooled LR + values obtained using a random-effect model among the markers (Table 2) revealed that for the HE4 maker, LR + was significantly higher than that of CA125 and ROMA. Using HE4, the likelihood of a woman diagnosed with EOC is about 11 times, given the woman test positive compared to a false positive woman. Further, LR + for HE4 is more than the recommended level (5.0), which was observed in the majority of the studies (84%) (Fig. 5A). Pooled LR + and it's 95% confidence limits (CI) for HE4, CA125 and ROMA were 10.61 (7.28–15.45), 2.91 (2.47–3.42) and 4.66 (3.41–6.35), respectively (Table 2). Only 11% of the studies with CA125 (Fig. 5B) showed LR + more than 5.0. Using ROMA marker, about 46% of the studies with premenopausal women (Fig. 5C) and 65% of the studies with postmenopausal women (Fig. 5D) showed LR + ve more than 5.0. Pooled LR-of all the three markers or algorithm were in the range between 0.18 and 0.27 (Table 2), implying that all the three were behaving similarly for ruling out the disease as evident by overlapping CI of the three markers or algorithm. Figure 6A–D shows the forest plots of LR- values using the random effect model for each marker.

**Figure 7:**
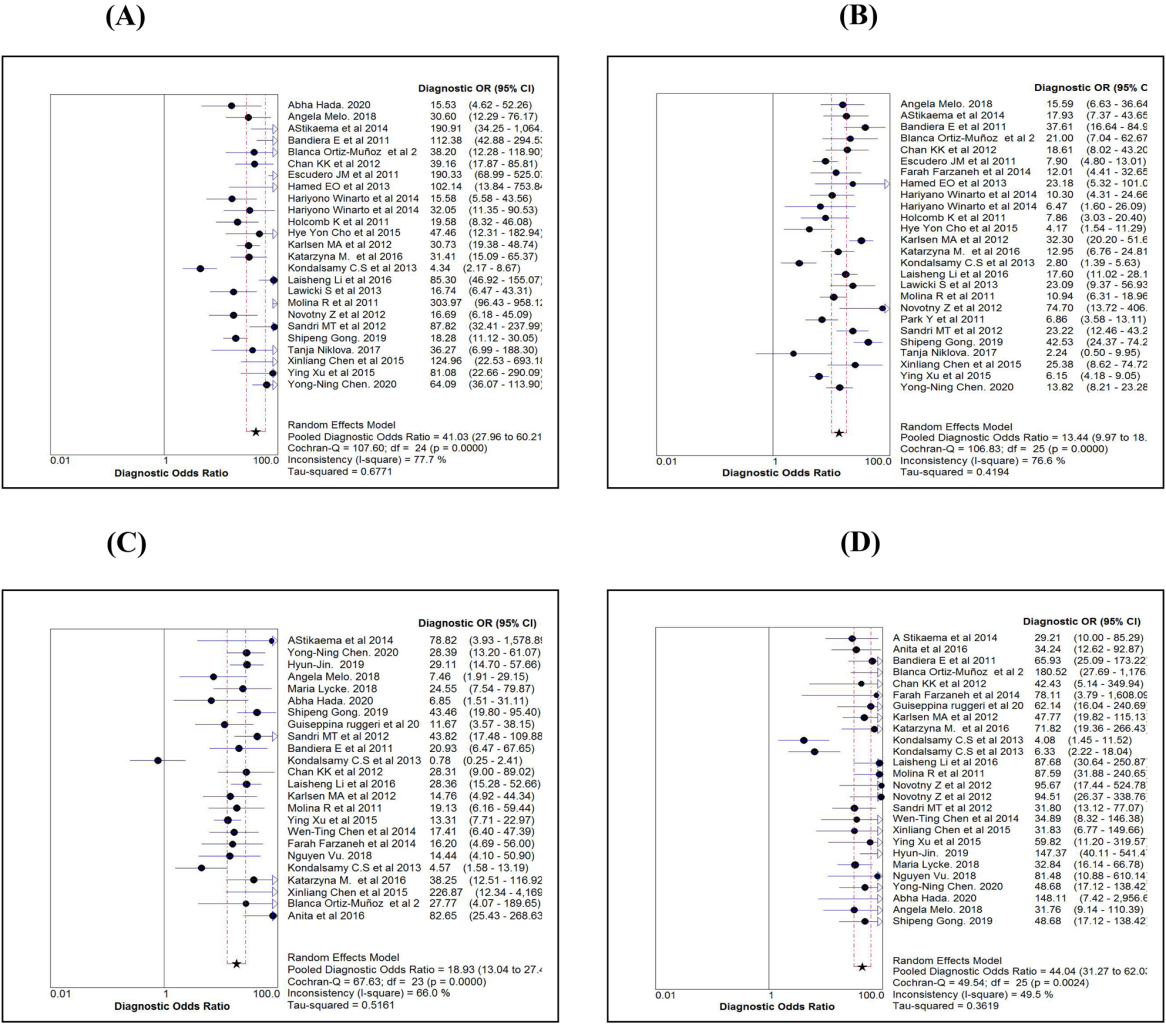
Comparison of diagnostic odds ratio estimates (using Random effect model) of HE4 (**A**), CA125 (**B**), ROMA for pre-menopause (**C**) and ROMA for post-menopause (**D**).

As accuracy estimates are paired and often interrelated (sensitivity and specificity, or LR positive and LR negative), it is necessary to report these simultaneously with a single measure. One accuracy measure that combines these paired measures is the diagnostic odds ratio (DOR). Individual study wise and pooled DOR with 95% CI for each marker are shown in Fig. 7A–D. Using the random effect model, pooled DOR for HE4 was 41.03 (95% CI 27.96–60.21), which was markedly higher compared to DOR of CA125 (13.44) and ROMA (27.48). Similarly, areas under the curve (AUC) using symmetrical ROC (SROC) analysis were 0.91, 0.86 and 0.91 for HE4, CA125 and ROMA, respectively (Table 2).

**Figure 8:**
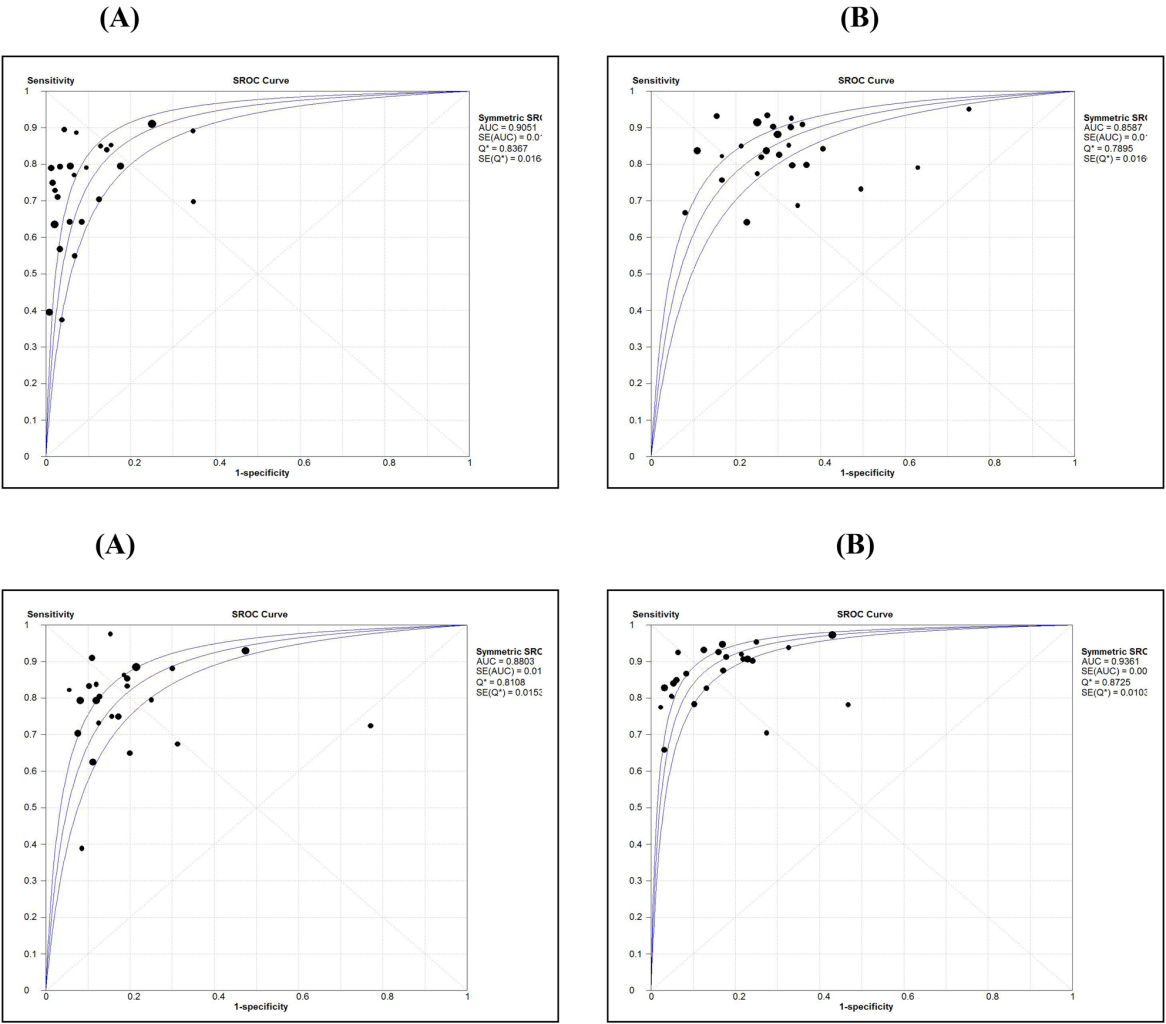
Comparison of Area Under Curve (AUC) by symmetric ROC analysis for HE4 (**A**), CA125 (**B**), ROMA for pre-menopause (**C**) and ROMA for post-menopause (**D**).

Test of heterogeneity for the markers or algorithm showed that the values of test statistics tau-square or I^2^-statistics were more significant than the allowable limit (50%), suggesting heterogeneity between the studies. Therefore, diagnostics measures obtained by the random effect model were retained. Spearman rank correlation coefficient between sensitivity and FPR showed that only for HE4, the correlation coefficient was significant (r = 0.49; p = 0.013) (Table 2). Regression analysis of diagnostic threshold also revealed that slope was significant (p < 0.05) for HE4 (Table 3). These observations showed that there was no evidence of a strong threshold effect on DOR. Further, the ROC curve (Fig. 8A–D) showed no clear evidence of a curve-linear pattern for any of the markers confirming that DOR measures were independent of the threshold effect.

**Table 3:** Results of meta regression analysis to assess causes of heterogeneity.

Variable	HE4	CA125	ROMA (ALL)	ROMA (pre-menopause)	ROMA (post-menopause)
**Analysis of diagnostic odds ratio (DOR)**					
Constant (SE)	3.27 (0.24)*	2.64 (0.19)*	3.35 (0.21)*	2.91 (0.20)*	3.85 (0.18)*
Slope (SE)	− 0.29 (0.11)*	− 0.06 (0.16)	− 0.10 (0.17)	− 0.17 (0.17)	− 0.17 (0.13)
Tau-square	0.50	0.42	0.75	0.57	0.40
**Meta regression with cutoff value as covariate#**					
Tau-square	0.36	0.45	0.84	0.64	0.46
Constant (SE)	1.31 (0.65)	2.76 (0.35)*	2.18 (1.60)	2.49 (0.88)*	3.67 (1.81)
S coefficient (SE)	− 0.14 (0.12)	− 0.14 (0.16)	− 0.03 (0.20)	− 0.12 (0.18)	− 0.16 (0.14)
Cutoff (SE)	0.02 (0.01)*	− 0.002 (0.01)	0.06 (0.08)	0.04 (0.08)	0.007 (0.06)
**Meta regression with age as covariate#**					
Tau-square	0.81	0.51	1.06	Could not be carried out due to non-availability of Age details separately for pre-menopause and post-menopause status	
Constant (SE)	2.57 (1.79)	1.60 (1.51)	1.63 (2.18)		
S coefficient (SE)	− 0.31 (0.16)	− 0.24 (0.19)	− 0.24 (0.25)		
Age (SE)	0.01 (0.03)	0.03 (0.03)	0.03 (0.04)		
**Meta regression with cutoff value and age as covariates#**					
Tau-square	0.49	0.78	1.30	Could not be carried out due to non-availability of Age details separately for pre-menopause and post-menopause status	
Constant (SE)	1.01 (1.57)	1.62 (2.93)	1.21 (2.65)		
S coefficient (SE)	− 0.18 (0.15)	− 0.30 (0.25)	− 0.15 (0.35)		
Cutoff (SE)	0.03 (0.01)*	0.02 (0.09)	0.05 (0.12)		
Age (SE)	− 0.003 (0.03)	0.013 (0.04)	0.02 (0.05)		

*Coefficients are statistically significant at P < 0.05, Human epididymis secretory protein 4 (HE4), Cancer antigen (CA125) and Risk of Ovarian Malignancy Algorithm (ROMA). Standard error (SE).

^#^Meta-regression was weighted by the inverse of the variance of diagnostic Odds Ratio and between study variance was estimated by Random Effect Model (REML).

### Meta-regression analysis to assess the source of heterogeneity

Since there was heterogeneity between the studies for the markers, we carried out meta-regression analysis to evaluate causes responsible for heterogeneity. Table 3 shows the results of the meta-regression analysis. The regression coefficient for the cutoff values of HE4 was statistically significant, showing the influence of cutoff value for the noted heterogeneity. However, neither age nor cutoff value was found to be statistically significant for CA125 and ROMA markers. Both threshold analysis and meta-regression analysis revealed the significant importance of cutoff value for HE4.

### Publication bias assessment

We assessed publication bias by carrying out a regression analysis of ln(DOR) against the inverse of the sample size's square root, and Table 4 shows the results. Slope coefficients for all the markers were not significant at p < 0.10, which is the probability level suggestive of significant publication bias. Therefore, there was no evidence of publication bias for any of the markers or algorithm.

**Table 4:** Statistical tests for publication bias by regression analysis of ln(DOR) against inverse of square root of effective sample size.

	Bias coefficient (SE)	P value	Intercept (SE)	P value
HE4	− 4.45 (7.46)	0.557	4.10 (0.56)	0.001
CA125	− 9.90 (5.79)	0.100	3.30 (0.44)	0.001
ROMA	− 15.30 (9.16)	0.110	4.32 (0.64)	0.001
ROMA-pre-menopause	0.24 (6.89)	0.973	3.00 (0.64)	0.001
ROMA-post-menopause	− 3.42 (4.99)	0.499	4.24 (0.51)	0.001

Human epididymis secretory protein 4 (HE4), Cancer antigen (CA125) and Risk of Ovarian Malignancy Algorithm (ROMA). Standard error (SE).

### Comparison of diagnostic measures between premenopausal and postmenopausal women

The majority of the studies reported test results separately for premenopausal and postmenopausal women for ROMA. A similar analysis for ROMA showed that the diagnostic measures such as sensitivity, specificity, LR + , DOR and SROC (Table 2) were markedly high for postmenopausal women than premenopausal women. LR was the lowest value (0.15) for postmenopausal women compared to the value (0.26) for premenopausal women. The threshold effect for DOR was not established, and heterogeneity was present for both groups. Cutoff value was not found to be an influencing factor for the observed heterogeneity. Since average age was not available separately, we could not carry out meta-regression to establish whether the age was influencing heterogeneity. Further, we could not observe publication biases for either type of study women.

### Sensitivity analysis

We carried out the sensitivity analysis to confirm the consistency of the results by considering only blinded studies, which were free from biased observations. The numbers of studies blinded were 17^16–18,23–29,32,35–41^, 19^16–18,20,23–29,32,33,35–41^ and 15^15–18,20,26–29,34,35,37–40^ for HE4, CA125 and ROMA, respectively. The meta-analysis of the blinded studies using the random-effect model is shown in Table 5. Comparing the results presented in Tables 2 and 5 showed that all the diagnostic measures such as sensitivity, specificity and DOR obtained for blinded studies were within 95% confidence limits of each measure presented in Table 2.

**Table 5:** Results of sensitivity analysis based on blinded studies using Random effect model.

Statistics (95% CI)	Name of marker		ROMA		
	HE4	CA 125	ROMA All	ROMA Pre-menopause	ROMA Post-menopause
Sensitivity	0.73 (0.71–0.75)	0.85 (0.83–0.87)	0.85 (0.83–0.87)	0.82 (0.79–0.85)	0.87 (0.85–0.89)
Specificity	0.89 (0.88–0.90)	0.70 (0.69–0.72)	0.75 (0.73–0.76)	0.75 (0.73–0.77)	0.81 (0.80–0.83)
LR + ve	9.37 (5.90–14.88)	2.61 (2.17–3.13)	4.39 (2.94–6.56)	4.19 (2.91–6.01)	5.56 (3.81–8.11)
LR –ve	0.26 (0.19–0.36)	0.22 (0.16–0.30)	0.18 (0.12–0.26)	0.23 (0.16–0.32)	0.16 (0.11–0.22)
Diagnostic odds ratio	37.88 (23.89–60.05)	12.38 (8.48–18.08)	25.70 (14.91–44.27)	19.44 (11.42–33.11)	38.21 (24.05–60.70)
Tau-square (%)	76.1	78.3	94.15	73.4	52.5
Cochrane-Q	66.94 (P = 0.001)	82.84 (P = 0.001)	105.26 (P = 0.001)	56.39 (P = 0.001)	40.46 (P = 0.001)
Spearman’s correlation	0.57 (P = 0.017)	− 0.07 (P = 0.778)	0.07 (P = 0.791)	0.07 (P = 0.791	0.35 (P = 0.171)
**Symmetrical ROC (SROC)**					
AUC (SE) Q* index	0.90 (0.01) 0.83 (0.02)	0.83 (0.03) 0..76 (0.03)	.91 (0.019) 0..84 (0.021)	0.89 (0.02) 0.82 (0.02)	0.93 (0.012) 0.86 (0.015)

Human epididymis secretory protein 4 (HE4), Cancer antigen (CA125) and Risk of Ovarian Malignancy Algorithm (ROMA). Positive Likelihood ratio (LR + ve), Negative Likelihood ratio (LR–ve), Standard error (SE), Receiver operating characteristic curve (ROC), Area under curve (AUC).

Further, the presence of heterogeneity was confirmed among blinded studies also. There was no significant variation in Spearman rank correlation coefficients and regression analysis of threshold effects between combined studies and blinded studies. We could make similar observations while carrying out meta-regression and SROC analysis also. These findings implied that all the diagnostic measures were consistent irrespective of blinding nature.

## Discussion

### Summary of diagnostic measures

Our study found ROMA as the best marker to differentiate EOC from benign ovarian masses with high diagnostic accuracy (DOR-44.04, sensitivity-0.88 and AUC-0.94) as compared to HE4 (DOR-41.03, sensitivity-0.73 and AUC-0.91) and CA125 (DOR-13.44, specificity-0.84 and AUC-0.86) in postmenopausal women. In premenopausal women, DOR, specificity and AUC for HE4 was highest, suggesting it as a promising predictor of Epithelial ovarian cancer in this group; however, its utilisation requires further exploration.

### Strengths

In this meta-analysis, we evaluated a good number of studies, which adds more weight to our results. Pertinent features of our study are the approach that has been used to search the articles and the statistical methods that are used to analyse the data. Other salient features of our study are that we had only included studies that used CLIA/ECLIA for measuring CA-125 and HE4, which reduced the bias that arise due to different testing strategies and also, we used only patients with EOC as cases and Benign ovarian masses as controls.

### Limitation

We did not consider unpublished and studies in foreign languages. Therefore, we could not calculate diagnostic measures for HE4 and CA125 in the premenopausal and postmenopausal group separately using the few studies. Further, we did not assess the diagnostic efficiency of these markers stratified by the stage and type of tumour because of the availability of only a few studies.

### Interpretation

The first meta-analysis to compare the diagnostic efficiency of HE4 compared to CA125 was published in January 2012 by Yu et al.^47^. They postulated that HE4 was a better marker than CA125 for sensitivity, specificity, LR + and LR−. Their study had methodological limitation like the inclusion of healthy subjects in the control group, and more than one index test was used. The pooling of such studies can cause increased variation among the studies. Later, Li et al.^48^, who did a meta-analysis based on 11 studies in 2012, stated that ROMA had high diagnostic accuracy in distinguishing EOC from benign pelvic masses. They also suggested that ROMA had higher sensitivity than HE4 and more accuracy in the postmenopausal age group than the premenopausal age group, as seen in our study. In 2014, Wang et al.^49^ also inferred similar findings and postulated that HE4 had more specificity than CA125 and could be of diagnostic importance, especially in the premenopausal group. A few months later, Macedo et al.^50^ published a meta-analysis and postulated that HE4 is a useful predictor of benign or malignant pelvic masses with AUC (0.91) similar to that seen in our study for premenopausal women. Substantiating our findings, a meta-analysis conducted by Liu et al. that included 17 studies also showed HE4 as a promising biomarker for endometrial cancer with high specificity, DOR and AUC^51^. A study conducted by Niveditha et al. proposed that preoperative low HE4 and biopsy has a high predictive value than preoperative MRI and intraoperative biopsy combined^52^. In another systematic review conducted by Degez et al., it was found that serum HE4 was a crucial parameter in assessing diagnosis, prognosis and survival of endometrial cancer^53^. Later in 2016, Dayyani et al.^54^ advanced that ROMA had higher diagnostic performance than other single marker assays based on the basis of AUC and would thus improve clinical decision making.

Ovarian cancer is the fifth most common cause for cancer deaths among women. It is very important to differentiate malignant and benign ovarian mass at an early stage as the risk of malignancy increases with age^55^. CA 125 has low sensitivity in early stages and also falsely elevated in non-malignant conditions. Whereas HE4 has higher specificity when compared to CA 125, but it is also increased in smokers and women who are on oral contraceptives^56,57^. On the other hand, ROMA has better diagnostic performance when compared to single marker assays and hence can be the diagnostic tool in EOC for postmenopausal women. In premenopausal women, HE4 is a better marker to differentiate malignant from benign ovarian masses.

## Conclusion

The study infers that ROMA is superior to HE4 and CA125in the postmenopausal group in distinguishing EOC from benign ovarian tumors. HE4 appears to be superior to CA125with diagnostic accuracy and prediction of EOC in the premenopausal group.
